# Influence of Natural Perlite Fineness Variation on the Physico-Mechanical Properties of Mortars

**DOI:** 10.3390/ma19214590

**Published:** 2026-10-29

**Authors:** Mustapha Lotfi Ghomari, Hamid Khelafi, El Hadj Kadri, Ramdane Chihaoui, Abdelwahhab Khatir

**Affiliations:** 1 LMST Laboratory, Civil Engineering Department, University of Sciences and Technologies Mohamed Boudiaf of Oran (USTO-MB), El-Mnaouer, Oran 31000, Algeria; 2 Faculté des Sciences et de la Technologie, Université Ahmed Draia, Adrar 01000, Algeria; ham.khelafi@univ-adrar.edu.dz; 3 Laboratoire L2MGC, Université de Cergy Pontoise, 95031 Cergy Pontoise, France; el-hadj.kadri@u-cergy.fr; 4 Laboratory LabMat, National Polytechnic School of Oran Morice Audin of Oran (ENPO), Bp 1523 EL M′NAOUER, Oran 31000, Algeria; rchihaoui@yahoo.fr; 5 LM2SC Laboratory, Civil Engineering Department, University of Sciences and Technologies Mohamed Boudiaf of Oran (USTO-MB), El-Mnaouer, Oran 31000, Algeria

**Keywords:** natural perlite, grinding, fineness, cement mortar, compressive strength, heat evolution, water-accessible porosity, superplasticizer demand

## Abstract

This study investigates the influence of natural perlite fineness on the physical and mechanical properties of cement mortars. Natural perlite was ground for 2, 4, and 6 h, producing Blaine fineness values of 3040, 5900, and 8030 cm^2^/g, respectively. Standardized mortars were prepared by replacing 15% and 25% of the cement mass with perlite. The effects of perlite fineness were evaluated through superplasticizer dosage, semi-adiabatic calorimetry, and compressive strength at curing ages from 1 to 180 days, and water-accessible porosity at 180 days. The results showed that perlite fineness influenced the fresh and hardened properties of the mortars. Increasing fineness generally reduced the superplasticizer dosage required to achieve the target consistency. Perlite-containing mortars exhibited lower early heat release than the reference mortar, and increasing perlite fineness partially enhanced heat evolution. The finest perlite improved compressive-strength development and produced the lowest water-accessible porosity at both replacement levels, particularly at later ages. These findings demonstrate that natural perlite fineness is an important parameter influencing the physical and mechanical properties of mortars.

## 1. Introduction

The continuous growth in the world population and the associated expansion of infrastructure and construction activities have contributed to a sustained increase in cement consumption and, consequently, in carbon dioxide emissions from the cement industry [1]. Global cement production is expected to remain at a very high level over the coming decades, making the reduction in the environmental impact associated with Portland cement an important challenge for the construction sector [2].

One of the strategies proposed to address this issue is the partial replacement of Portland cement with supplementary cementitious materials (SCMs), particularly naturally occurring pozzolanic materials. The use of such materials can reduce the clinker content of cement-based materials while taking advantage of their physical and chemical contributions to the cementitious matrix [3,4]. Depending on their nature, fineness, mineralogical composition, and reactivity, these materials can also modify the rheological behavior, mechanical performance, and durability of cement-based materials [5,6,7]. The flow and transport behavior of cementitious slurries are also strongly influenced by particle characteristics, rheological properties, and fluid–particle interactions, as demonstrated in recent studies of cement-based slurry systems [8].

Several studies have demonstrated the potential of natural pozzolans as partial replacements for Portland cement. For example, improved long-term mechanical performance has been reported for mortars and concretes containing natural pozzolanic materials, particularly at moderate replacement levels [9,10]. More generally, the mechanical performance of cement-based materials is closely related to changes in their internal microstructure and porosity, which can evolve substantially under different environmental and thermal conditions [11]. Natural perlite has also attracted attention because of its relatively high silica content and its availability in several regions. Previous studies conducted on Algerian perlite have confirmed its potential pozzolanic character [5,6]. Furthermore, as highlighted by Chihaoui et al. (2022) [12], the incorporation of natural perlite produces low-Ca/Si ratio C-S-H phases that exhibit an enhanced capacity for alkali (Na^+^ and K^+^) binding, effectively depleting the alkali concentration in the pore solution. Combined with the consumption of free Ca(OH)^2^ required for gel swelling, the refined microstructure limits moisture ingress, thereby preventing the expansive pressure associated with ASR gel formation in the cementitious matrix. However, despite its high SiO_2_ content, natural perlite may exhibit relatively low reactivity in its untreated state. Consequently, its incorporation at relatively high replacement levels can result in a reduction in mechanical strength, particularly at early ages [5,6].

Different approaches have been investigated to modify the properties of natural perlite for use in cement-based materials, including thermal treatment, chemical treatment, and grinding [13,14]. Grinding is of particular interest because it can reduce particle size and increase specific surface area without requiring high-temperature treatment or additional chemical reagents. These changes in fineness may influence particle packing, the interaction between particles and the cementitious matrix, and potentially the dissolution behaviour of reactive constituents. However, the extent to which these physical and chemical effects contribute to mortar performance depends on the material characteristics and the treatment conditions.

Motahari et al. [13,15] investigated the effect of further milling of natural perlite used as a supplementary cementitious material. Perlite powders with different fineness levels, ranging from approximately 3100 to 3900 cm^2^/g, were incorporated at replacement levels of 10% and 15%. Their results indicated that additional grinding could improve the mechanical performance of perlite-containing mixtures. At 90 days, mortars incorporating 10% finely ground perlite achieved strengths comparable to those of the reference mortar.

The combined use of mechanically activated perlite and nanosilica was subsequently investigated by Motahari et al. [16]. Their results showed that mechanical activation increased the potential reactivity of perlite, while the incorporation of nanosilica further promoted the development of the cementitious matrix. In particular, the combination of finely ground perlite and a small amount of nanosilica resulted in substantial improvements in mechanical performance.

Erdem et al. [17] investigated the use of natural perlite in blended cement systems prepared with Blaine fineness values of approximately 3200 and 3700 cm^2^/g and perlite replacement levels of 20% and 30%. Although the incorporation of perlite generally reduced early-age strength compared with the reference cement, the finer perlite produced better mechanical performance, which was attributed to its enhanced pozzolanic contribution.

The beneficial effect of grinding is not limited to perlite. Burris and Juenger [18], for example, demonstrated that milling natural zeolites can increase both their physical filler contribution and their pozzolanic reactivity, leading to improved mechanical performance of cement-based materials.

Previous studies (Table 1) have investigated the use of natural perlite as a supplementary cementitious material, including its influence on cement and mortar properties and the effects of milling on its performance [5,6,13,17,19]. However, limited information is available on the combined influence of fineness variation and replacement level on overall performance of perlite-containing mortars. In particular, the relationships between perlite fineness, superplasticizer demand, heat evolution, long-term strength development, and pore structure require further investigation. Consequently, a systematic evaluation is needed to determine the extent to which an increase in the replacement level and fineness of perlite affects the behavior of the mortar, both in the short term and long term.

The objective of the present study is to investigate the influence of natural perlite fineness on the physico-mechanical properties of cement mortars. Three fineness levels were examined, corresponding to grinding durations of 2, 4, and 6 h and Blaine fineness values of 3040, 5900, and 8030 cm^2^/g, respectively. The perlite was incorporated at cement replacement levels of 15% and 25%. Its influence on superplasticizer dosage, heat evolution, compressive strength development from 1 to 180 days, and water-accessible porosity was assessed. This study focuses on the relationships between the measured fineness and the properties of the resulting mortars; it does not directly quantify pozzolanic activity or independently separate physical filler effects from chemical contributions.

## 2. Materials and Methods

### 2.1. Materials

A Portland cement CEM I 52.5 conforming to the Algerian standard NA 442 [20] was used throughout the experimental program. Its chemical and mineralogical compositions, together with its main physical properties, are presented in Table 2. The cement had a density of 3.20 g/cm^3^ and a Blaine specific surface area of 5130 cm^2^/g. The clinker phase contents were estimated using the Bogue equations from the cement oxide composition.

**Figure 1:**
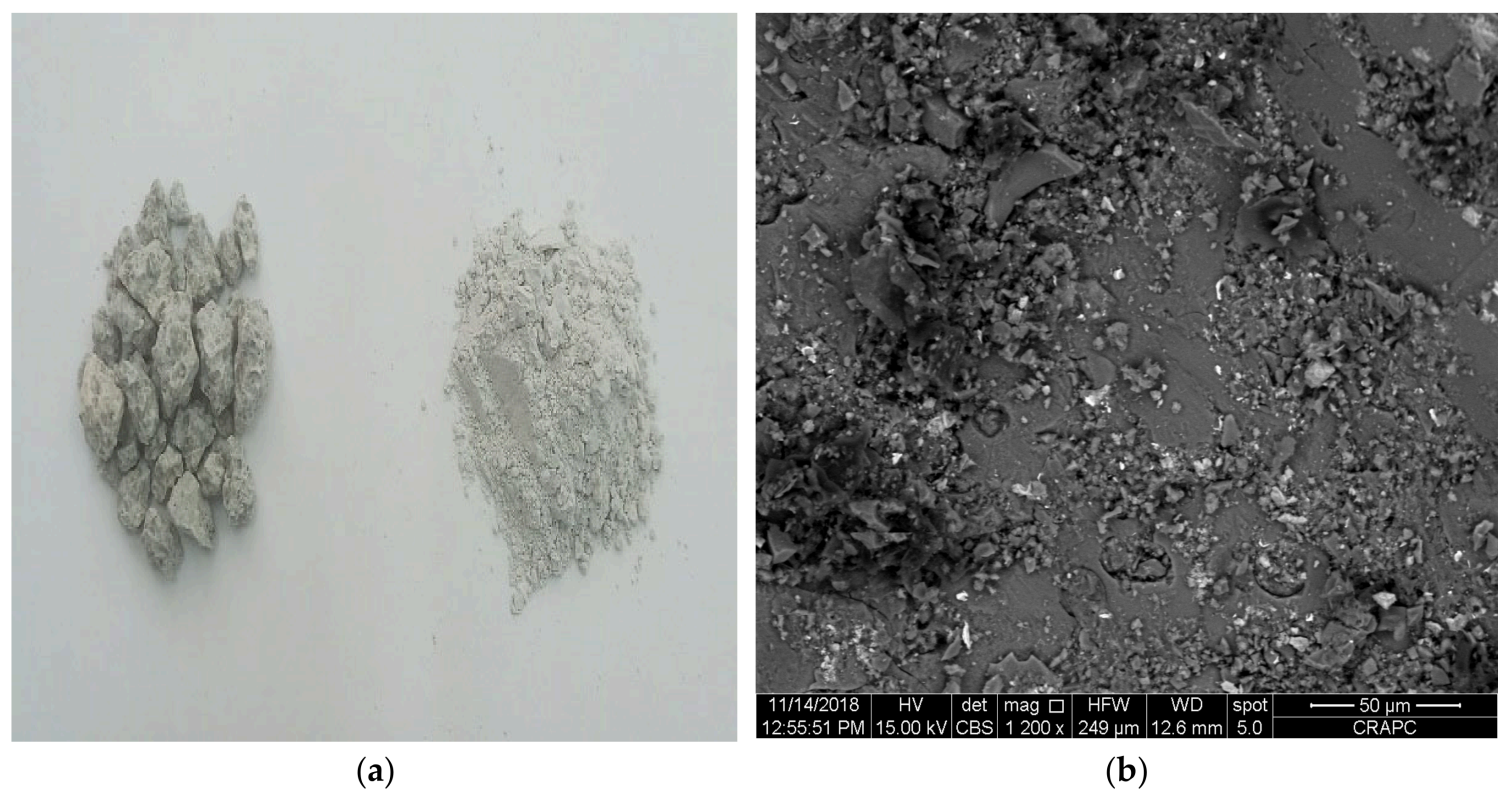
Natural perlite from Hammam Boughrara region, Algeria: (**a**) natural perlite before and after grinding; (**b**) SEM image of natural perlite.

The mineral addition investigated in this study was natural perlite obtained from deposits located in western Algeria (Figure 1). Its chemical composition and physical properties are summarized in Table 3.

Prior to grinding, the natural perlite was crushed into coarse particles with diameters ranging from approximately 15 to 25 mm. The amount of crushed perlite to be ground was 6 kg; this amount was kept constant throughout the grinding process. The grinding was carried out using a laboratory grinder measuring 40 cm in length and 55 cm in diameter. This vertical-shaft grinder operated at a rotational speed of 30 rpm. The charge of the grinding mill consisted of steel spherical balls. The total weight of the charge was 70 kg. The crushed perlite was subsequently ground for 2, 4, and 6h to obtain three different fineness levels, corresponding to Blaine values of 3040, 5900, and 8030 cm^2^/g, respectively.

**Figure 2:**
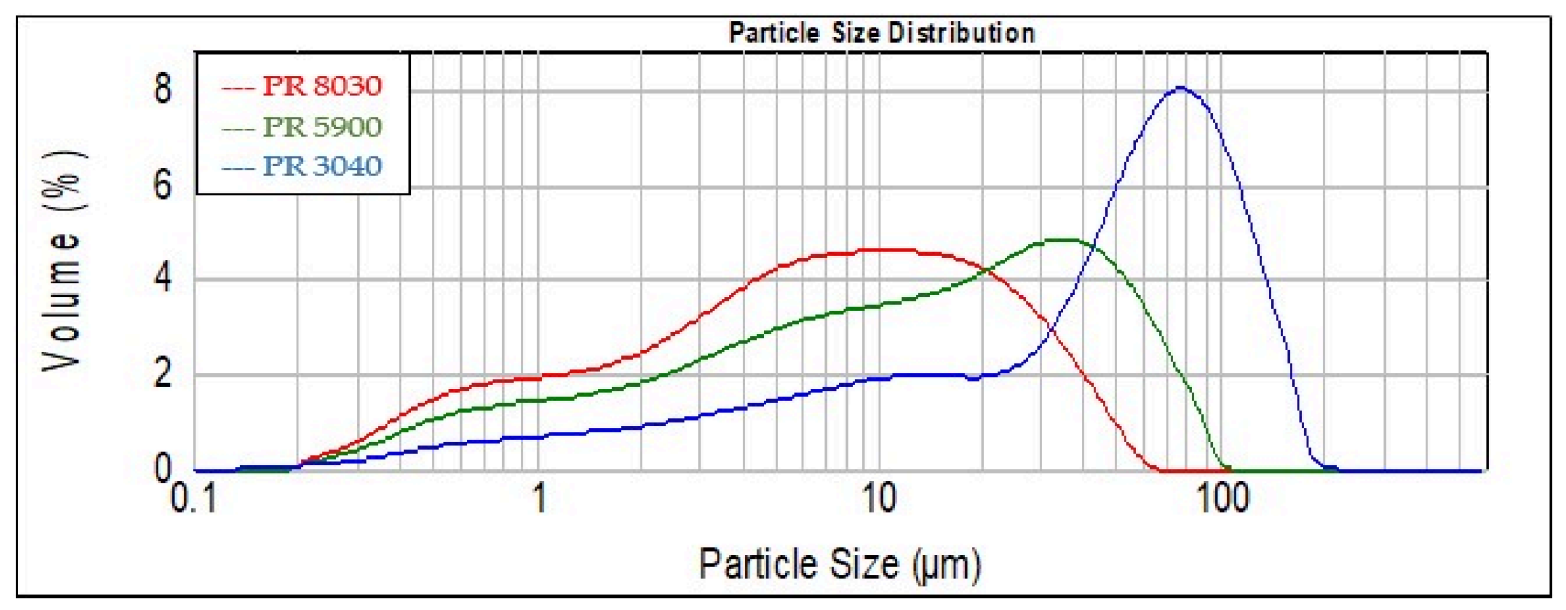
Particle-size distributions of perlite powders with different fineness.

The fineness of the ground perlite was characterized using the Blaine air permeability method. The values obtained are used in this study as operational indicators of fineness. They should not be interpreted as direct measures of the true specific surface area, as the Blaine response can be influenced by particle morphology, irregular shape, and internal porosity. In the absence of BET measurements, the contributions of accessible surface area and internal porosity could not be quantified separately. The particle size distribution data were therefore considered as supplementary information for evaluating the effect of grinding. The particle-size distributions of the three perlite powders, determined by laser granulometry, are presented in Figure 2.

The particle-size distributions showed a clear shift toward finer particle sizes with increasing Blaine fineness. The values of D10, D50, and D90 are presented in Table 4. These results confirm the substantial reduction in particle size associated with prolonged grinding, particularly for PR 8030.

A polycarboxylate-based high-range water-reducing admixture was used in all mixtures. Its main characteristics are listed in Table 5.

A standardized sand conforming to EN 196-1 [21] was used to prepare all mortars. Each batch contained 1350 ± 5 g of standardized sand.

### 2.2. Mortar Mixtures

The mortars were prepared according to EN 480-1 [22], using two cement replacement levels, namely 15% and 25% by mass, with natural perlite. Mixing was performed in a vertical-shaft mixer with a capacity of 5 L. The total mixing time was 3 min and 30 s. Regarding the preparation of (40 × 40 × 160 mm^3^) test specimens, the mortar was compacted into the mold by pouring it in two layers and tapping the mold 60 times after each layer. The mold was then trimmed and leveled, covered with plastic wrap, and stored in a room at 20 °C where the humidity was 90%.

The three fineness levels were obtained by varying the grinding time. PR 3040 represented the coarsest powder, obtained after 2 h of grinding, whereas PR 5900 and PR 8030 corresponded to intermediate and high fineness levels obtained after 4 and 6 h of grinding, respectively. The effect of perlite fineness on the superplasticizer demand was evaluated by determining the dosage, as a percentage of the binder mass based on the liquid product, required to achieve a mortar spread of 17 cm, with an acceptance tolerance of ±5%. The superplasticizer dosage was progressively adjusted in 0.05% increments by mass of binder. This test involves measuring the spread diameter of mortar prepared with different superplasticizer dosages using a flow table, in accordance with EN 413-2 [23]. Although the water-to-cement ratio increased from 0.40 for the reference mortar to approximately 0.47 and 0.53 for the mortars containing 15% and 25% perlite, respectively, this increase results from calculating the ratio using the remaining Portland cement content. The water-to-binder ratio remained constant at 0.40 because the total binder content and mixing-water content were kept constant. The liquid content of the superplasticizer was not included in the water content of the W/B ratio. The increased water-to-cement ratio should nevertheless be considered when interpreting the strength development, since the availability of water relative to the remaining clinker may influence its hydration. Accordingly, the observed strength development cannot be attributed exclusively to the pozzolanic contribution of perlite. Seven mortar mixtures were investigated: one ordinary reference mortar and six perlite-containing mortars incorporating three perlite fineness levels at two replacement percentages. The resulting mixture proportions are summarized in Table 6.

Since cement and perlite have different densities (3.20 and 2.46 g/cm^3^, respectively), mass-based replacement at a constant total binder mass results in an increase in the absolute volume of the solid binder. The 450 g reference binder corresponds to an absolute volume of 140.63 cm^3^. This volume increases to 146.97 and 151.20 cm^3^ for the mixtures containing 15% and 25% perlite by mass, respectively, corresponding to increases of approximately 4.5% and 7.5% relative to the reference mixture. On a volumetric basis, the 15% and 25% mass replacements correspond to approximately 18.7% and 30.3% replacement of the binder phase, respectively.

### 2.3. Experimental Procedures

**Figure 3:**
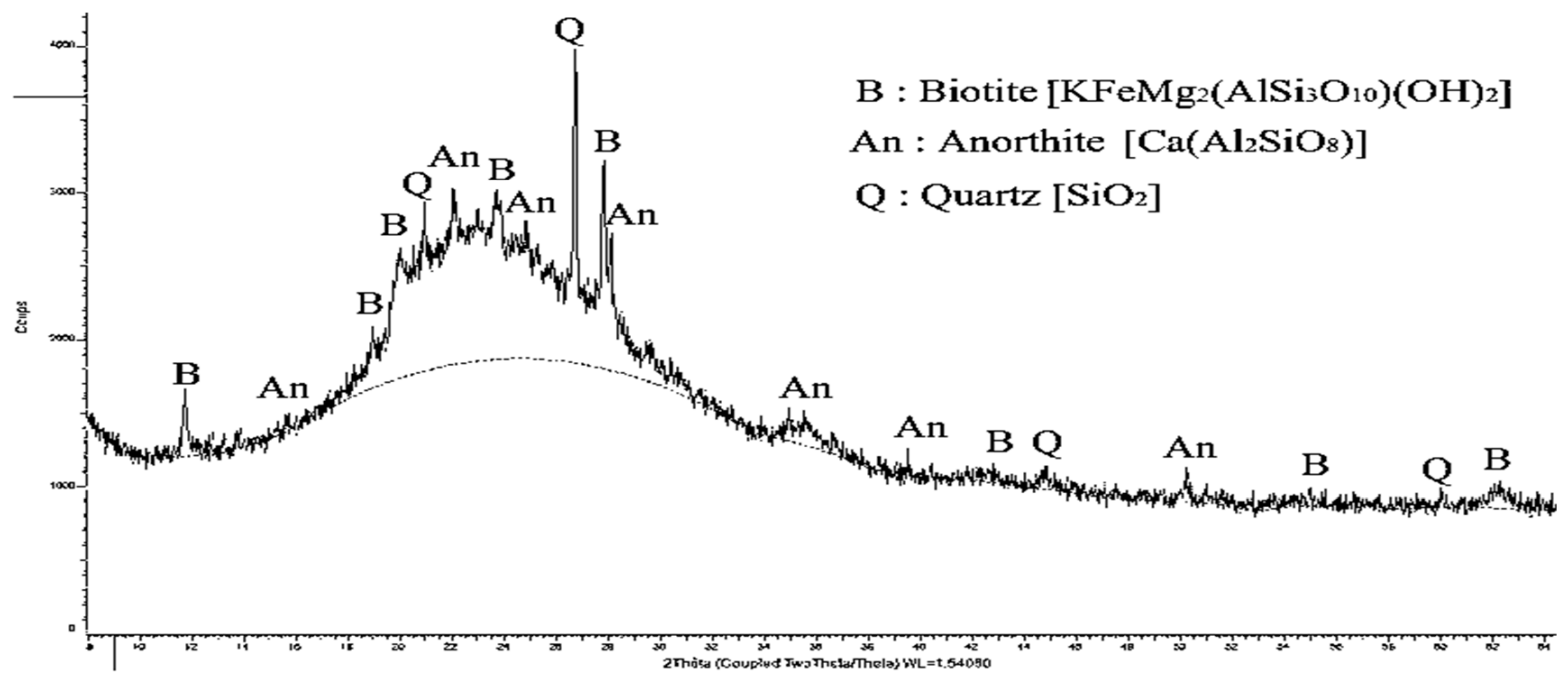
XRD pattern of natural perlite PR 3040.

X-ray diffraction (XRD) analysis was performed to characterize the mineralogical composition of the natural perlite and identify its crystalline and amorphous components. XRD analysis was performed using a Bruker D8 Advance diffractometer equipped with *Cu Kα*, and approximately *λ* = 1.5406 Å. The diffraction patterns were collected over a *2 θ* range of 8–65°, using a step size of 0.02°. The resulting patterns were used to identify the crystalline phases present in the examined powder. The XRD pattern of the ground perlite is presented in Figure 3. The effect of perlite fineness and replacement level on the required superplasticizer dosage was evaluated by determining the saturation dosage necessary to achieve the target mortar consistency. The results were compared with those obtained for the reference mortar.

The hydration heat of the mortars was monitored using semi-adiabatic calorimetry according to EN 196-9 [24]. The measurements were performed during the first 72 h after mixing. The reference mortar contained 450 g of Portland cement, whereas the perlite-containing mortars contained a total binder mass of 450 g, with natural perlite replacing 15% or 25% of the cement by mass. Accordingly, the Portland cement content was 382.5 g and 337.5 g for the mortars containing 15% and 25% perlite, respectively. The water content was 180 g for all mixtures, corresponding to a water-to-binder ratio of 0.40. A superplasticizer was used to achieve the target mortar consistency.

The cumulative heat release, (q), was expressed in J/g of binder, normalized to the mass of Portland cement and perlite present in each mixture. Therefore, the results should be interpreted in the context of the different binder compositions and replacement levels. Because the measured heat evolution reflects the behaviour of the complete mortar mixtures, this normalization does not independently quantify the heat contribution of perlite or establish its pozzolanic activity. The heat-release values were calculated using Equation (1):

(1) $$q=\frac{C}{m_{b}}.\Delta \theta +\frac{1}{m_{b}}\int_{0}^{t}\alpha .\Delta \theta .dt$$

where *q* is the cumulative heat release normalized to the mass of binder, *C* is the total thermal capacity of the calorimeter, *m_b_* is the binder mass defined as the sum of Portland cement and perlite (g), *Δθ* is the temperature increase of the testing mortar at time *t*, *t* is the duration of hydration *(h)*, and *α* is the total calorimeter thermal loss coefficient (J/h °C).

Compressive strength tests were conducted on three prismatic specimens (40 × 40 × 160 mm^3^) in accordance with NF EN 196-1 [21]. The specimens were demolded 24 h after casting and then kept in water at 20 °C until the test age. The test ages considered were 1, 3, 7, 28, 90, and 180 days. The compression test is performed by applying a loading rate of 2400 N/s to the test specimen. Each compressive strength value was calculated as the average of the results obtained from three test specimens. Accordingly, a total of 126 specimens were used for the compressive-strength program (7 mixtures × 3 specimens × 6 curing ages).

Water-accessible porosity was determined according to NF P18-459 [25]. Each value was calculated as the average of the results obtained from three test specimens (40 × 40 × 160 mm^3^). The porosity, ε, expressed as a percentage of the specimen volume, was calculated using Equation (2).

(2) $$\varepsilon \;(\%)=\frac{Mair-Mdry}{Mair-Mwater}\;\times \;100$$

where M_water_ is the mass of the water-saturated specimen determined by hydrostatic weighing, M_air_ is the mass of the specimen in air after hydrostatic weighing, and M_dry_ is the mass of the specimen after drying at 105 ± 5 °C.

## 3. Results and Discussion

### 3.1. X-Ray Diffraction Analysis of Natural Perlite

The XRD pattern of the natural perlite after grinding is presented in Figure 3.

The XRD analysis was used to characterize the mineralogical features of the examined natural perlite powder. The diffraction pattern showed crystalline peaks superimposed on a broad diffuse halo approximately between 18° and 30° 2θ, indicating the coexistence of crystalline and amorphous components. The proposed crystalline-phase assignments should be considered in conjunction with the bulk chemical composition, since the presence of a diffraction peak alone does not establish the quantitative abundance of a particular mineral. Similarly, the qualitative XRD pattern does not allow reliable quantification of the crystalline and amorphous fractions. Although amorphous aluminosilicate constituents may participate in reactions under the alkaline conditions of cement hydration, the presence of an amorphous halo alone does not establish the extent of pozzolanic activity. Moreover, comparative XRD measurements and quantitative amorphous-phase analyses were not available for all three perlite fineness levels. Therefore, the present results cannot establish whether grinding altered the relative proportions of crystalline and amorphous phases or the degree of structural disorder. Accordingly, the XRD results are used to describe the mineralogical characteristics of the examined powder and are not considered direct evidence of pozzolanic activity or grinding-induced structural activation. The influence of grinding is instead discussed in terms of the measured Blaine fineness and the observed mortar properties, without assuming that particle size or specific surface area was the only material characteristic affected.

### 3.2. Effect of Perlite Fineness on Superplasticizer Demand

**Figure 4:**
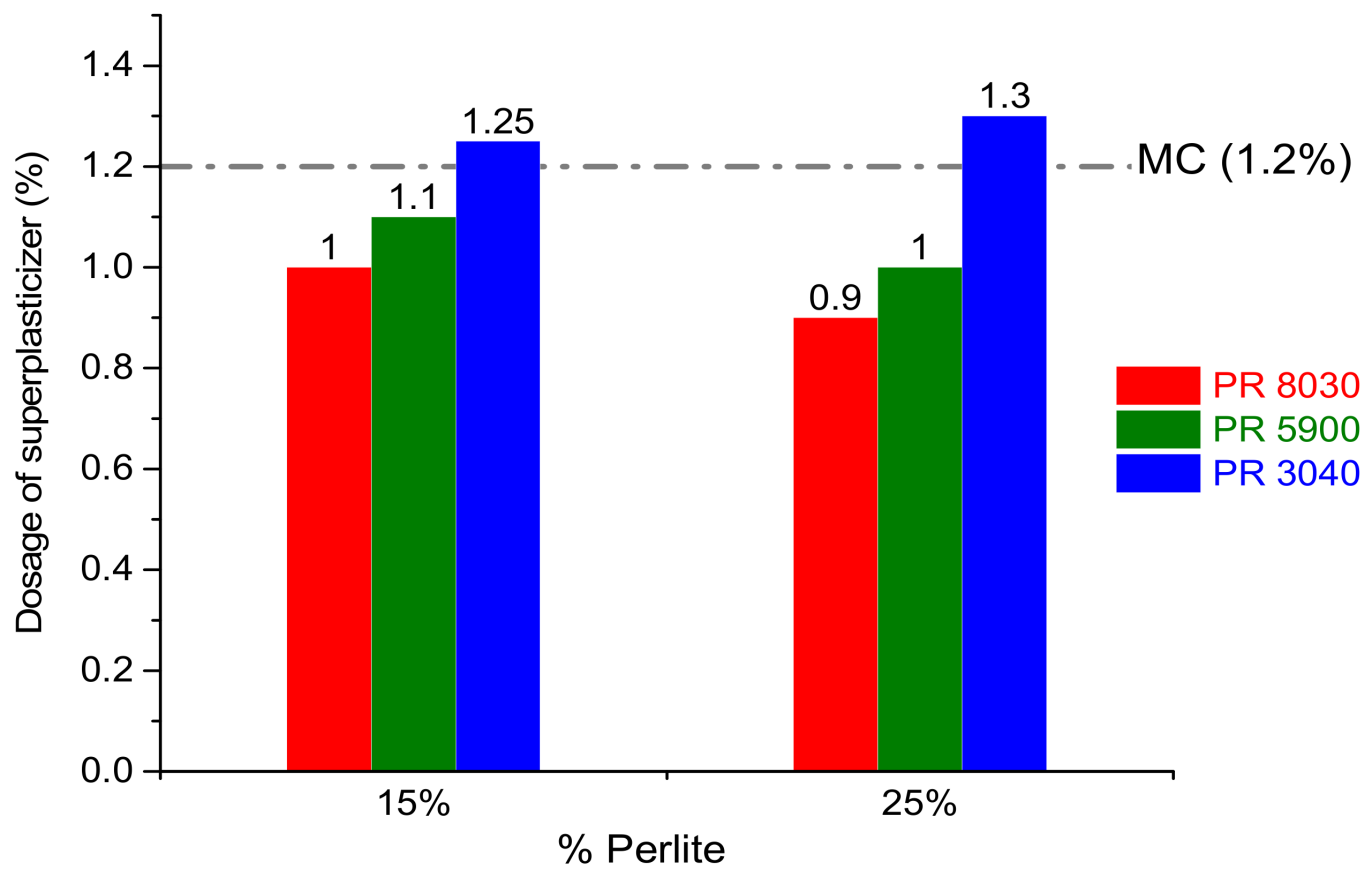
Effect of perlite replacement level and fineness on the superplasticizer dosage.

Figure 4 presents the dosage of superplasticizer demand to achieve the target mortar consistency as a function of perlite replacement level and fineness.

A clear difference can be observed between the three perlite powders. Mortars incorporating the finer powders, PR 8030 and PR 5900, required lower superplasticizer dosages than the reference mortar at both replacement levels. In contrast, the PR 3040 mixture required a dosage comparable to that of the reference mortar at a 15% replacement level and a slightly higher dosage at 25%. This behavior can be related to the modification of the particle packing and dispersion state of the cementitious system resulting from the incorporation of finely divided perlite. Fine particles can occupy interstitial spaces between cement grains, thereby modifying the packing structure and reducing the amount of water physically trapped within particle flocs. This can improve the effective availability of water and consequently reduce the amount of superplasticizer required to achieve the target consistency [26,27]. Previous studies have shown that particle-size distribution plays an important role in the rheological behavior of cementitious suspensions. The presence of fine particles can increase packing density and modify the balance between interparticle forces and liquid availability [28,29]. Grzeszczyk [30] also reported that fine mineral particles can modify the response of cementitious systems to polycarboxylate-based superplasticizers. In the present study, the behavior of PR 3040 can be attributed to its relatively coarse particle size and lower specific surface area. Compared with the finer powders, the less refined particle-size distribution may result in less efficient packing and greater water retention within the particle network. Consequently, a higher superplasticizer dosage may be required, particularly at the 25% replacement level. These results indicate that grinding does not simply increase the surface area of perlite; it also changes its interaction with the cementitious suspension and consequently its rheological demand.

### 3.3. Heat of Hydration

**Figure 5:**
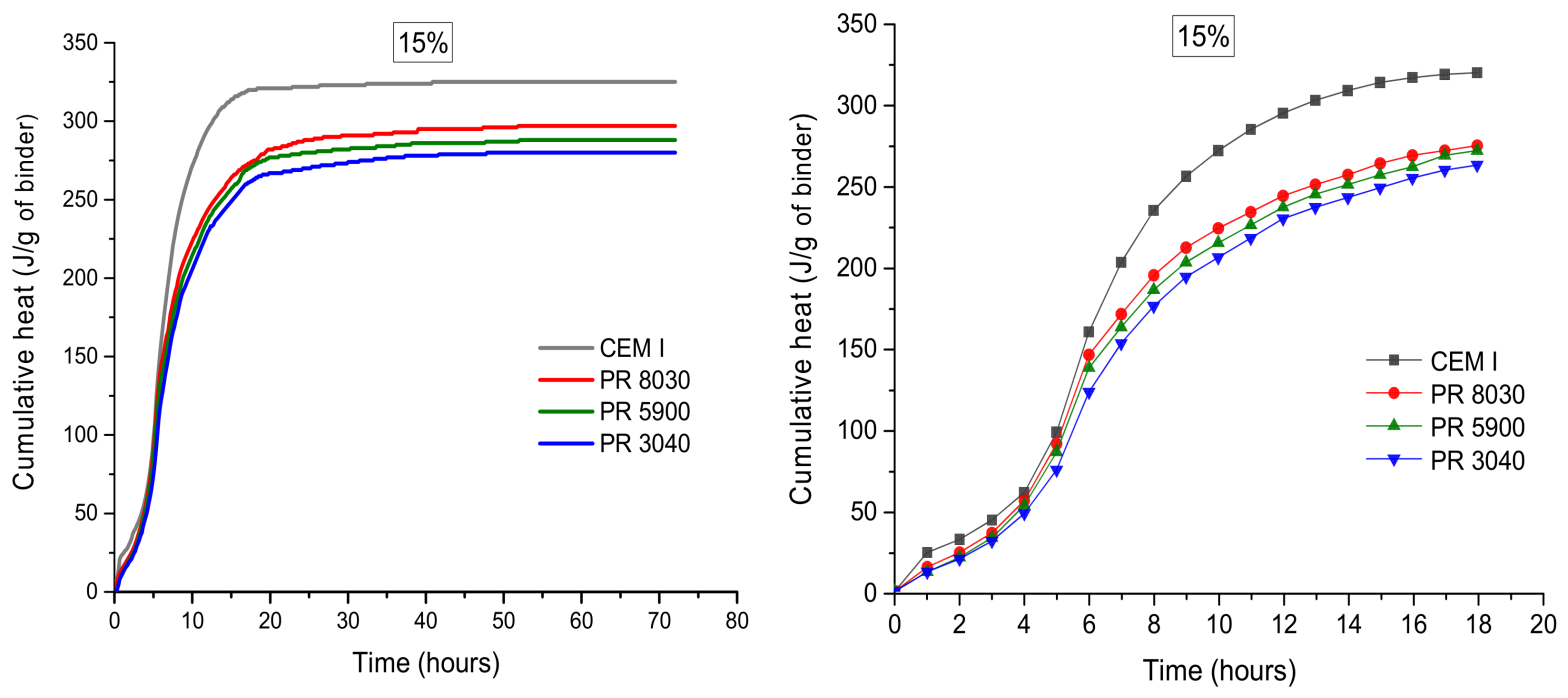
Heat evolution of mortars containing 15% natural perlite with different fineness levels compared with the reference mortar.

**Figure 6:**
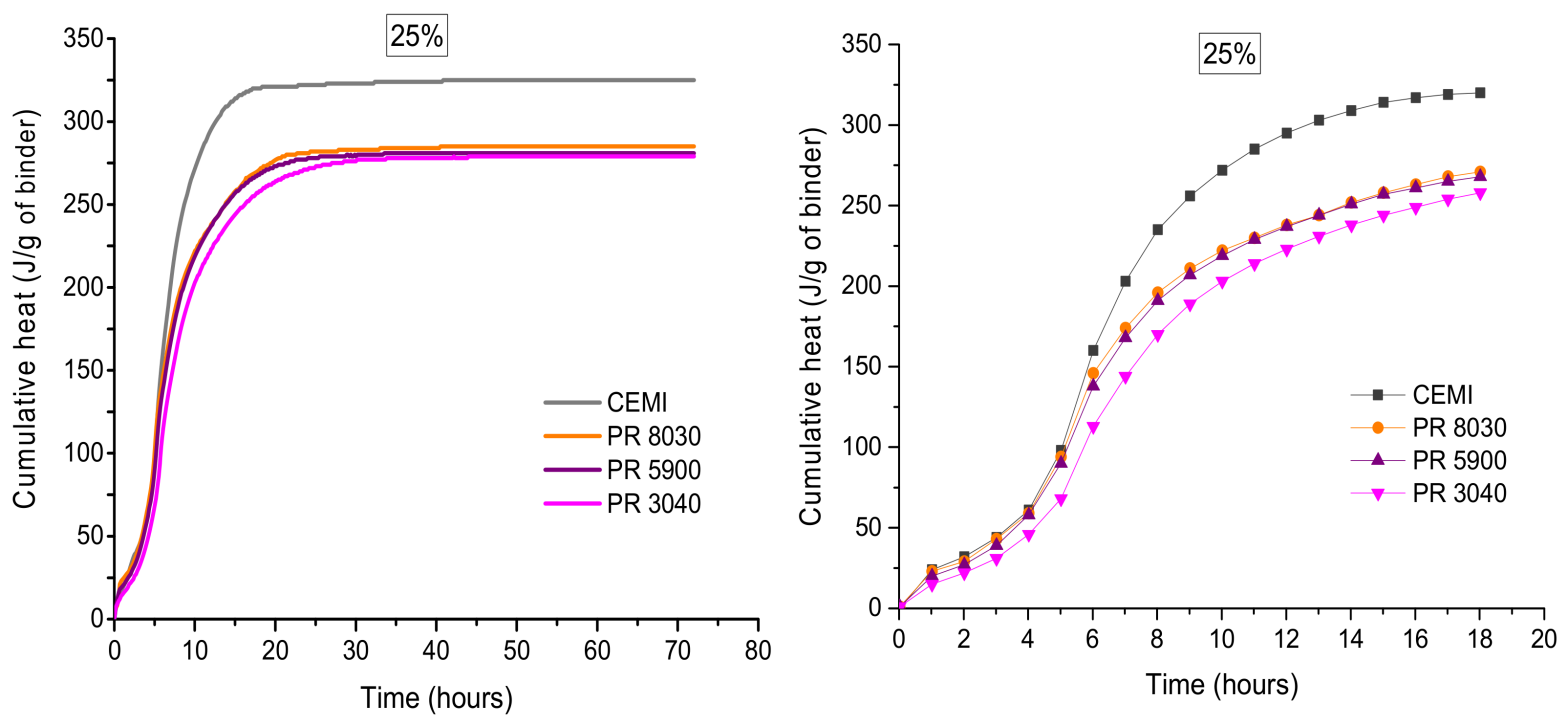
Heat evolution of mortars containing 25% natural perlite with different fineness levels compared with the reference mortar.

The heat evolution curves obtained during the first 72 h of hydration are presented in Figure 5 and Figure 6.

The reference mortar exhibited the most rapid hydration kinetics and the highest cumulative heat release. Approximately 83% of its final heat release was reached after about 10 h, increasing to approximately 90% after 12 h. This behavior is consistent with the relatively high C_3_S content of the Portland cement and, importantly, its relatively high Blaine fineness of 5130 cm^2^/g. The high specific surface area of the cement can promote rapid hydration and contribute to the pronounced early heat release observed for the reference mortar. Although C_3_A also contributes to early hydration reactions, its content in the present cement is relatively modest (5.69%); therefore, the rapid early-age heat development is more appropriately associated with the combined effect of the cement mineralogical composition, particularly its C_3_S content, and its high fineness [31].

The partial replacement of Portland cement with natural perlite resulted in lower cumulative heat release per gram of binder than the reference mortar. Because the cumulative heat values are normalized to the mass of binder, this difference cannot be attributed simply to the lower total amount of Portland cement in the mixture. Instead, it reflects the combined thermal behavior of the mortar mixtures under the adopted testing conditions. The higher effective water-to-cement ratios of the perlite-containing mixtures, differences in particle packing and surface characteristics, superplasticizer dosage, and possible contributions from reactions involving perlite may all influence the measured heat evolution. However, the present calorimetry measurements do not allow these individual effects to be separated or the extent of any pozzolanic contribution to be quantified. In addition, the pozzolanic reaction of natural perlite develops more slowly than the hydration of Portland cement and therefore contributes less to heat release during the early stages of hydration. Similar behavior has been reported for blended cement systems containing pozzolanic materials [5,31]. The replacement level also affected the heat evolution. Mortars containing 15% perlite generally released more heat than those containing 25%, as expected from the higher amount of cement available for hydration. For example, the cumulative heat release reached approximately 297 and 288 J/g for PR 8030 and PR 5900, respectively, at a 15% replacement level, compared with approximately 285 and 281 J/g at a 25% replacement level.

Because the cumulative heat release is expressed per gram of binder, the effect of cement replacement cannot be interpreted solely as a simple dilution effect. Although the amount of Portland cement decreases with increasing perlite replacement, the effective water-to-cement ratio increases from 0.40 for the reference mortar to approximately 0.47 and 0.53 for the mixtures containing 15% and 25% perlite, respectively. This provides greater water availability per unit mass of cement and may facilitate cement hydration. In addition, the finely divided perlite provides additional solid surfaces that may act as nucleation sites for hydration products. The effect becomes more pronounced for the finer PR8030, suggesting that increased fineness enhances the surface-mediated contribution of perlite to the hydration process. Therefore, the measured heat release per gram of binder reflects not only clinker dilution but also the combined influence of water availability, particle surface effects, and the potential contribution of perlite reaction.

It should also be noted that the present measurements were obtained using semi-adiabatic calorimetry. Consequently, the temperature experienced by each mixture was not strictly identical during the test, and the results should not be interpreted as equivalent to those obtained under isothermal calorimetry. A more detailed comparison of hydration kinetics would require heat-flow measurements under controlled isothermal conditions. Nevertheless, the present results provide useful comparative information on the effect of perlite fineness under the adopted semi-adiabatic testing conditions.

It should nevertheless be emphasized that heat evolution alone cannot directly quantify the extent of the pozzolanic reaction, as the measured thermal behaviour reflects the combined effects of cement hydration, clinker dilution due to perlite replacement, and the physical characteristics of the perlite particles, including their potential influence on particle packing. The observed differences therefore indicate changes in the overall hydration kinetics of the mixtures.

The calorimetry measurements were conducted under the adopted semi-adiabatic testing procedure without replicate measurements for each mixture. Therefore, small differences in cumulative heat release between mixtures, particularly differences of only a few J/g, should not be over interpreted as statistically significant differences. The calorimetry results are consequently used primarily to identify the overall effect of cement replacement and perlite fineness rather than to establish the significance of very small differences between individual mixtures.

### 3.4. Compressive Strength

**Figure 7:**
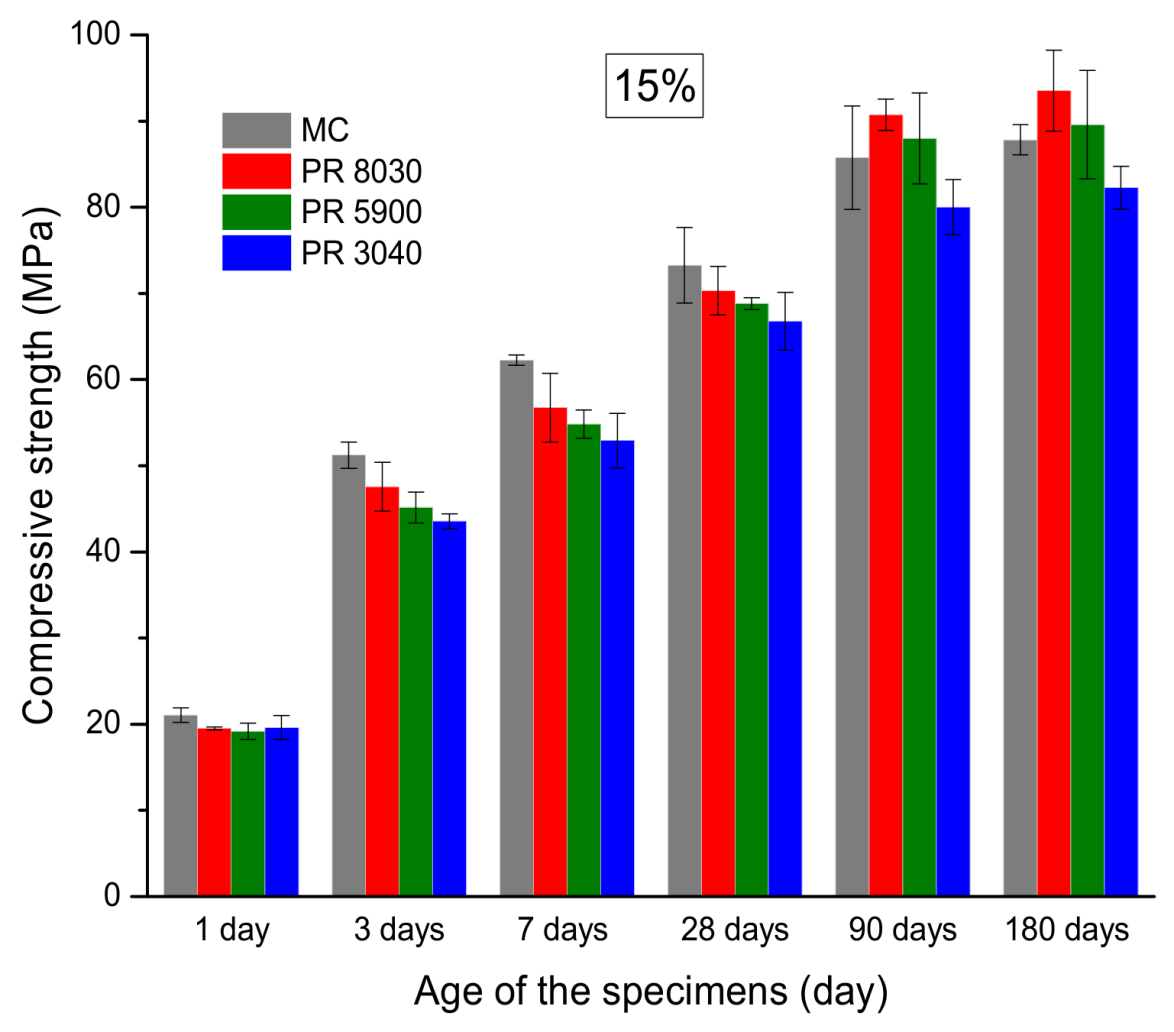
Effect of perlite fineness on the compressive strength of mortars containing 15% of perlite. The plotted values represent the average compressive strength of three specimens for each mixture and curing age.

**Figure 8:**
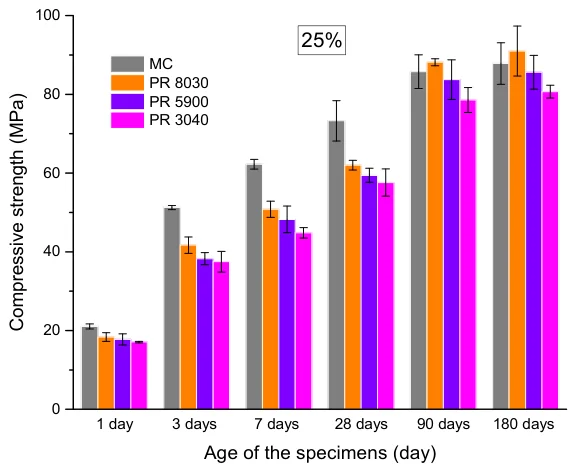
Effect of perlite fineness on the compressive strength of mortars containing 25% perlite. The plotted values represent the average compressive strength of three specimens for each mixture and curing age.

**Figure 9:**
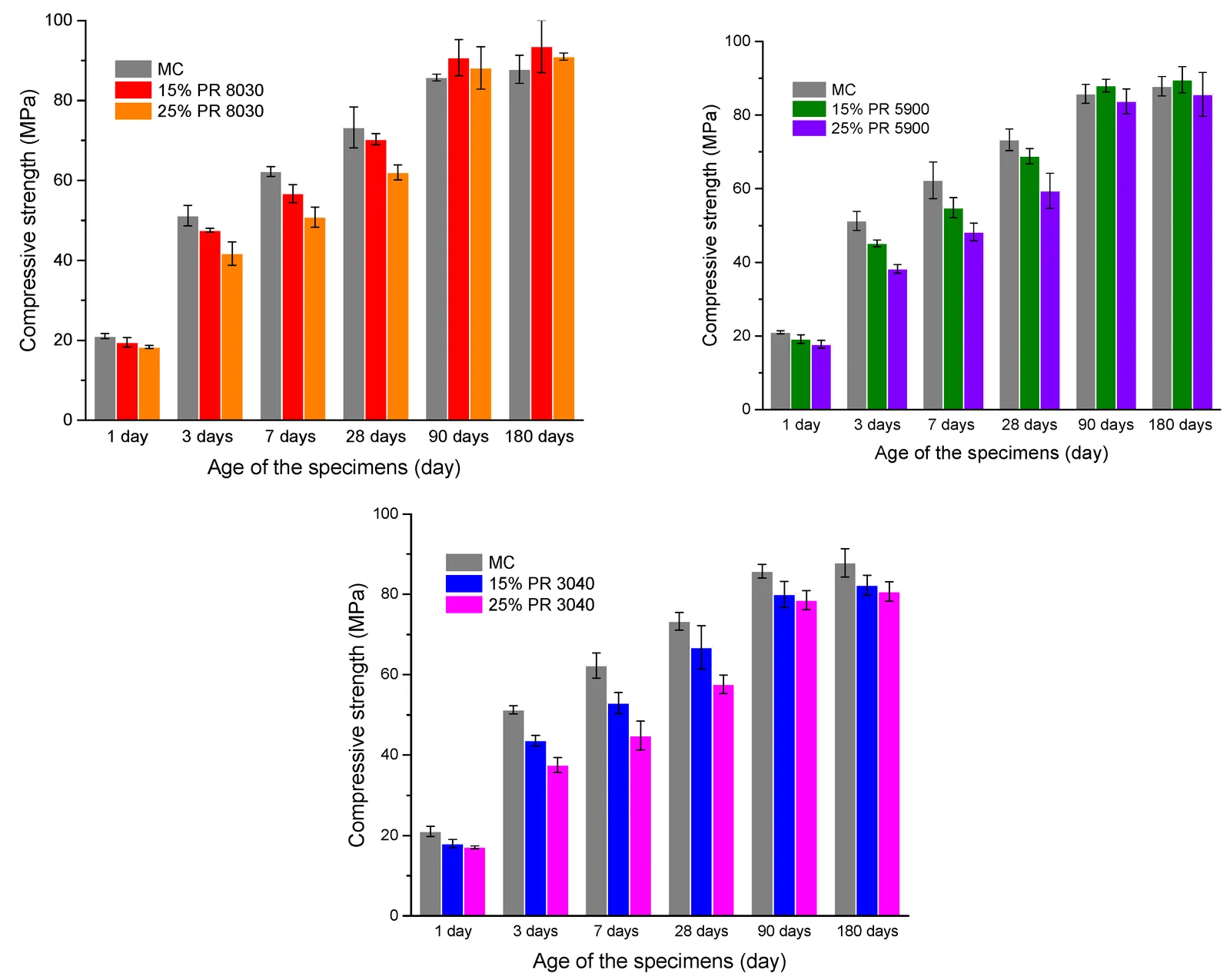
Evolution of compressive strength for mortars containing 15% and 25% perlite with the same fineness. The plotted values represent the average compressive strength of three specimens for each mixture and curing age.

The development of compressive strength as a function of curing age is shown in Figure 7, Figure 8 and Figure 9.

At early ages, all mortars containing perlite exhibited lower compressive strengths than the reference mortar. The strength reduction was generally more pronounced at the 25% replacement level than at the 15% level. At 1 day, the compressive strengths of the mortars containing perlite ranged from 19.15 to 19.60 MPa at 15% replacement, and from 17.09 to 18.33 MPa at 25% replacement, compared with 21.04 MPa for the reference mortar. These reductions are consistent with the lower Portland cement content and the resulting dilution effect. The heat evolution results also showed differences between the reference and perlite-containing mixtures; however, these measurements do not independently establish the contribution of pozzolanic reactions or physical effects. The influence of perlite fineness was also evident at early ages, although the differences among fineness levels were relatively small at 15% replacement and more noticeable at 25% replacement. As curing progressed, the strength differences among the perlite-containing mortars became more pronounced, with the finer perlite generally producing higher compressive strengths at a given replacement level. This trend is consistent with previous observations concerning the beneficial effect of mineral-addition fineness on strength development [32].

At 28 days, the reference mortar still exhibited the highest compressive strength. However, the strength gap between the reference and perlite-containing mortars decreased as the perlite became finer. This behavior suggests that the increased specific surface area progressively enhanced the contribution of the perlite to the developing microstructure. At later ages, particularly at 90 days and beyond, the differences between the mixtures became substantially smaller. Some mortars containing finely ground perlite achieved strengths comparable to, or even higher than, that of the reference mortar. The later-age strength development may be associated with physical and chemical contributions from the finer perlite, potentially including reactions involving reactive constituents; however, these mechanisms were not directly measured in the present study.

The results obtained at 180 days further emphasize the importance of perlite fineness. Mortars containing 25% perlite, particularly PR 8030 and PR 5900, developed compressive strengths comparable to those containing 15% perlite. This behavior indicates that the lower amount of clinker in the 25% mixtures can be partially compensated for by the greater long-term contribution of the finely ground perlite.

At 180 days, the mortars containing perlite with a Blaine fineness of 8030 cm^2^/g reached average compressive strengths of 93.54 MPa and 91.00 MPa at replacement levels of 15% and 25%, respectively, compared with 87.84 MPa for the reference mortar. In contrast, the corresponding strengths of the mortars containing perlite with a fineness of 3040 cm^2^/g were 82.29 MPa and 80.69 MPa. These results illustrate the influence of perlite fineness on long-term compressive strength at both replacement levels. However, the reported values are averages, and the differences are interpreted descriptively rather than as statistically significant, since statistical significance has not been established.

The comparison between replacement level and fineness is particularly interesting. At long curing ages, the use of 25% PR 8030 allowed the mortar to reach or exceed the strength obtained with coarser perlite at a lower replacement level. This observation highlights the strong interaction between replacement level and particle fineness. A higher replacement level does not necessarily lead to proportionally lower long-term performance when the added perlite possesses sufficient reactivity.

The strength development therefore reflects the combined influence of the volumetric effect of perlite replacement, particle-size refinement, packing characteristics, and possible chemical contributions from the perlite. The beneficial effect observed at later ages for the finer perlite powders is consequently interpreted as a combined effect rather than as a sole consequence of increased finer particle-size distribution. However, because no inert powder with comparable fineness was included as a control, the respective contributions of physical and chemical mechanisms cannot be separated quantitatively in the present study. The observed strength development and activity-index trends are therefore consistent with a possible contribution from pozzolanic reactions but do not constitute independent proof of their extent.

To further compare strength development across curing ages, the compressive-strength activity index was calculated as the ratio of the average compressive strength of each perlite-containing mortar to that of the reference mortar at the same age, expressed as a percentage, as illustrated in Table 7. At 28 days, all perlite-containing mortars exhibited activity indices below 100%. By 90 days, the indices of the mortars containing perlite with a fineness of 8030 cm^2^/g reached 105.8% and 102.8% at replacement levels of 15% and 25%, respectively. The corresponding values at 180 days were 106.5% and 103.6%. For the 5900 cm^2^/g perlite, the activity index exceeded 100% at 90 and 180 days at the 15% replacement level, whereas it remained below 100% at the 25% replacement level. The mortars containing perlite with a fineness of 3040 cm^2^/g remained below the reference mortar throughout the test period. These results indicate that the relative long-term strength development depended on both perlite fineness and replacement level. The increase in activity index at later ages is consistent with a possible contribution from pozzolanic reactions; however, it cannot independently distinguish chemical effects from physical effects, clinker dilution, or differences associated with the water-to-cement ratio.

From a practical perspective, the finest perlite investigated (8030 cm^2^/g) was associated with the highest later-age compressive strength among the perlite-containing mixtures at both replacement levels. However, this finding identifies the most favorable fineness among those investigated in terms of later-age strength, rather than an overall optimum. The energy consumed during grinding was not measured, and the available data therefore do not permit calculation of the specific grinding energy or assessment of the associated environmental impact. A comprehensive evaluation of the optimum fineness would require measured grinding-energy consumption together with strength, durability, and cost considerations. Accordingly, 8030 cm^2^/g is identified as the most favorable of the investigated fineness levels for later-age compressive strength, but not as a demonstrated energy-efficient or environmentally optimal value.

The interpretation of the effects of perlite fineness should also consider the differences in superplasticizer dosage among the investigated mixtures. As reported in Table 6, the dosage required to achieve the target mortar consistency ranged from 0.9% to 1.3%, depending on the perlite fineness and replacement level. Consequently, the observed differences in compressive strength and water-accessible porosity cannot be attributed exclusively to perlite fineness, because fineness and superplasticizer dosage varied together. Although the results show an association between perlite fineness and mortar performance, the individual contributions of fineness and superplasticizer dosage cannot be isolated from the present experimental design.

### 3.5. Water-Accessible Porosity

**Figure 10:**
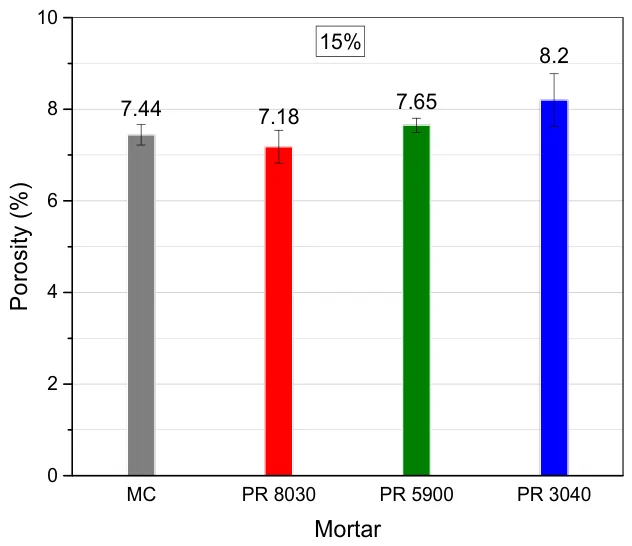
Water-accessible porosity of mortars containing 15% perlite at 180 days.

**Figure 11:**
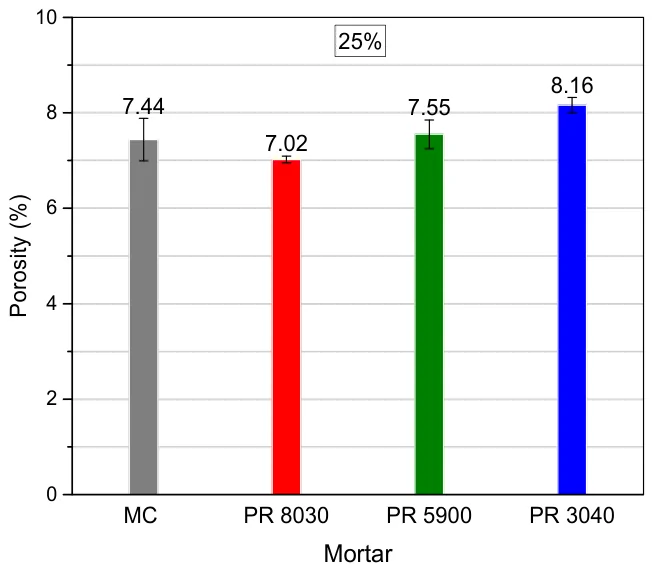
Water-accessible porosity of mortars containing 25% perlite at 180 days.

The water-accessible porosity measured at 180 days is presented in Figure 10 and Figure 11 for mortars containing 15% and 25% perlite, respectively. The reference mortar exhibited a water-accessible porosity of 7.44%, whereas the mortar containing perlite with a Blaine fineness of 8030 cm^2^/g (PR 8030) exhibited a value of 7.18%. This difference indicates a modest reduction in water-accessible porosity for PR 8030 compared with the reference mortar. The observed differences among the mixtures indicate variations in the volume of pores accessible to water. These differences may be associated with several factors, including particle-size distribution, particle packing, cement replacement level, differences in binder volume, and hydration-related processes. However, the present water-accessible porosity measurements cannot separate these effects or establish a specific physical or chemical mechanism responsible for the observed differences.

The porosity results should be interpreted in conjunction with the compressive-strength results. Although perlite fineness influenced mortar performance, the relationship between water-accessible porosity and compressive strength is not necessarily direct. Strength depends on several factors, including the properties of the hardened matrix and the interfaces between its constituents. Differences in porosity among the mixtures may be associated with the combined effects of perlite fineness, cement replacement, particle packing, and hydration-related processes; however, the relative contributions of these factors cannot be determined from the present measurements alone.

Water-accessible porosity quantifies the open pore volume accessible to water but does not provide pore-size distribution, pore connectivity, or direct information about transport properties. Consequently, the measurements do not establish pore refinement or independently demonstrate the occurrence or extent of pozzolanic reactions. Moreover, as porosity was measured only at 180 days, its evolution at earlier curing ages could not be evaluated. The results are therefore interpreted as differences in water-accessible porosity among the investigated mortars, rather than as direct evidence of a specific physical or chemical mechanism.

## 4. Conclusions

This study investigated the influence of natural perlite fineness variation on the physico-mechanical properties of cement mortars containing 15% and 25% perlite as a partial replacement for Portland cement. The principal conclusions are as follows:

Grinding increased the Blaine fineness of the natural perlite from 3040 cm^2^/g after 2 h to 5900 cm^2^/g after 4 h and 8030 cm^2^/g after 6 h. The resulting fineness variation was associated with changes in the fresh and hardened properties of the mortars.

Perlite fineness influenced the superplasticizer dosage required to achieve the target consistency. The finer powders generally required lower dosages than the coarser powder, although the response depended on the replacement level.

The incorporation of perlite resulted in lower cumulative heat release per gram of binder than the reference mortar. Increasing perlite fineness partially enhanced heat evolution, suggesting a contribution from surface-mediated nucleation and the probable pozzolanic reaction of the finely ground perlite.

Compressive strength generally increased with perlite fineness at a given replacement level. Although perlite-containing mortars exhibited lower strengths at early ages, at later ages, some mixtures containing finer perlite reached or exceeded the reference mortar strength. The compressive strength activity index demonstrates improved later-age strength development for the finest perlite investigated.

Water-accessible porosity decreased with increasing perlite fineness, with PR 8030 producing the lowest porosity, particularly at the high replacement level. This behavior indicates differences in water-accessible porosity among the investigated mixtures and may be associated with differences in particle packing and other physical or chemical effects; however, the present measurements do not allow these mechanisms to be separated or confirmed.

Overall, the results demonstrate that natural perlite fineness is an important parameter influencing the physico-mechanical properties of cement mortars. However, because the study did not include a direct pozzolanic-activity test or an inert-powder control, the findings do not establish the extent of the pozzolanic reaction or quantitatively distinguish it from physical filler effects and clinker dilution. Further characterization would be required to isolate these contributions. In addition, the variation in superplasticizer dosage among the mixtures represents a potential confounding factor, and its contribution to the observed differences in mortar performance could not be separated from the effect of perlite fineness.

## Figures and Tables

**Table 1:** Previous studies on perlite in cement-based materials.

Study	Perlite Fineness	Replacement Level	Relevant Measured Outcomes
Erdem et al. (2007) [17]	Blended cement Blaine fineness of 320 and 370 m^2^/kg; these are cement/blend values, not directly equivalent to perlite-powder fineness	20% and 30%	Blended-cement properties and compressive strength
Motahari et al. (2018) [13]	Two ground perlite powders with different specific surface areas: coarser and finer perlite	Multiple replacement ratios	Compressive strength and transport-related properties
Wang et al. (2024) [19]	Waste perlite powder (WPP)	5%, 10%, 20%, and 30%, rather than changing the fineness of the same perlite powder	Mortar/paste mechanical properties and material characterization

**Table 2:** Chemical and physical properties of the cement.

Chemical Composition (%)	Portland Cement
CaO	63.83
SiO_2_	22.41
Al_2_O_3_	4.21
Fe_2_O_3_	3.23
MgO	2.39
SO_3_	2.98
K_2_O	0.46
Na_2_O	0.10
LOI	3.46
Mineralogical composition (%)	
C_3_S	48.09
C_2_S	21.57
C_3_A	5.69
C_4_AF	9.82
Physical properties	
Density	3.20
Blaine fineness (cm^2^/g)	5130

**Table 3:** Chemical composition and physical properties of natural perlite.

Chemical Composition (%)	Natural Perlite
CaO	1.43
SiO_2_	72.54
Al_2_O_3_	14.78
Fe_2_O_3_	2.26
MgO	0.16
SO_3_	0.01
K_2_O	3.88
Na_2_O	1.72
LOI	3.99
Physical properties	
Density	2.46
Blaine fineness, PR 8030 (cm^2^/g)	8030
Blaine fineness, PR 5900 (cm^2^/g)	5900
Blaine fineness, PR 3040 (cm^2^/g)	3040

**Table 4:** Characteristic particle sizes of the ground perlite powders.

Powder	D10 (µm)	D50 (µm)	D90 (µm)
PR 8030	0.83	6.72	27.00
PR 5900	1.14	12.63	51.84
PR 3040	2.91	49.67	115.19

**Table 5:** Main characteristics of the superplasticizer.

Property of Superplasticizer	Characteristics
Appearance	Liquid
Color	Brown
Density	1.06 ± 0.03
Solid content	26 ± 1.3%
pH	7.1
Chloride content	<0.1%

**Table 6:** Mixture proportions of the investigated mortars.

Mortar	Perlite (%)	Binder (g)	Sand (g)	Cement (g)	Perlite (g)	Water (g)	W/B	W/C	SP (%)
MC	0	450	1350 ± 5	450	0	180	0.4	0.4	1.2
PR 8030-15	15	450	1350 ± 5	382.5	67.5	180	0.4	0.47	1
PR 8030-25	25	450	1350 ± 5	337.5	112.5	180	0.4	0.53	0.9
PR 5900-15	15	450	1350 ± 5	382.5	67.5	180	0.4	0.47	1.1
PR 5900-25	25	450	1350 ± 5	337.5	112.5	180	0.4	0.53	1
PR 3040-15	15	450	1350 ± 5	382.5	67.5	180	0.4	0.47	1.25
PR 3040-25	25	450	1350 ± 5	337.5	112.5	180	0.4	0.53	1.3

**Table 7:** Compressive-strength activity index of perlite-containing mortars relative to the reference mortar.

Curing Age	15% PR 8030	15% PR 5900	15% PR 3040	25% PR 8030	25% PR 5900	25% PR 3040
1 day	92.6%	91.0%	93.2%	87.1%	84.4%	81.2%
3 days	92.8%	88.1%	85.0%	81.4%	74.6%	73.2%
7 days	91.1%	88.1%	85.0%	81.6%	77.5%	72.0%
28 days	96.0%	93.9%	91.1%	84.6%	81.1%	78.6%
90 days	105.8%	102.6%	93.3%	102.8%	97.6%	91.6%
180 days	106.5%	102.0%	93.7%	103.6%	97.5%	91.9%

## Data Availability

The original contributions presented in this study are included in the article. Further inquiries can be directed to the corresponding authors.
